# Human Diseases Associated with Form and Function of the Golgi Complex

**DOI:** 10.3390/ijms140918670

**Published:** 2013-09-10

**Authors:** Mariana G. Bexiga, Jeremy C. Simpson

**Affiliations:** School of Biology and Environmental Science & Conway Institute of Biomolecular and Biomedical Research, University College Dublin, Dublin 4, Ireland; E-Mail: mariana.bexiga@ucd.ie

**Keywords:** Golgi complex, intracellular trafficking, membrane traffic, protein glycosylation, disease

## Abstract

The Golgi complex lies at the heart of the secretory pathway and is responsible for modifying proteins and lipids, as well as sorting newly synthesized molecules to their correct destination. As a consequence of these important roles, any changes in its proteome can negatively affect its function and in turn lead to disease. Recently, a number of proteins have been identified, which when either depleted or mutated, result in diseases that affect various organ systems. Here we describe how these proteins have been linked to the Golgi complex, and specifically how they affect either the morphology, membrane traffic or glycosylation ability of this organelle.

## 1. Introduction

Higher eukaryotic cells have evolved a highly compartmentalized endomembrane system in order to both increase efficiency and diversity of the biochemical processes that they can perform. In order to ensure that the correct complement of proteins and lipids necessary to maintain cellular homeostasis are present in each organelle, highly discrete membrane trafficking pathways connecting the various membrane-bounded organelles exist. Disruption of specific transport steps between the endoplasmic reticulum (ER), Golgi complex, endosomal-lysosomal system and the plasma membrane all can have dramatic consequences on the cell, and increasingly defects in the molecular machinery regulating membrane traffic are being linked to hereditary diseases [1,2].

In mammalian cells the Golgi complex has a distinct architecture [3,4], which at the ultrastructural level is characterized by a series of long ribbon-like membranes tightly opposed to one another. At the macro level this organelle is usually positioned centrally in the cell, close to the nucleus, and as such is considered as having a juxta-nuclear localization. In addition to its physical location at the center of the cell, this organelle also lies at the heart of the trafficking routes running through the endomembrane system. In the case of the secretory pathway it both receives newly synthesized proteins from the ER, and subsequently exports these same proteins to the endosomal-lysosomal system and the cell surface. During transit through the Golgi complex key modifications are made to most proteins, including changes to their glycosylation profile, sulfation, phosphorylation, and also proteolytic cleavage. As such, if Golgi homeostasis is perturbed then this is likely to affect its function, which in turn may result in disease. Several Golgi-related diseases have been identified, but the last few years have seen an increase in the identification of novel disease-related genes. Here, we focus our attention on several of these genes, and discuss the major phenotypes with respect to the Golgi complex: trafficking, morphological changes, glycosylation defects and finally loss-of-function of Golgi-residents.

## 2. When Traffic Comes to a Halt

Due to its central role in the secretory pathway, it is expected that any changes to the proteome of the Golgi complex would affect its homeostasis and consequently the flux of proteins trafficking through it. In recent years several diseases have been linked in various ways with trafficking at the Golgi, both in terms of receiving material from the ER, and also in the retrograde direction recycling components back to the ER. However, disentangling whether the transport defect is anterograde or retrograde is often difficult as both pathways are highly dependent on each other. One recent example of a molecule affecting transport at this level is the proteolipid protein 1 (*PLP1*), which is one of the main components of myelin. Mutations in *PLP1* have been connected to a wide spectrum of hypomyelinating disorders such as Pelizaeus-Merzbacher disease [5]. In this disease, mutant PLP1 is misfolded and accumulates in the ER, which in turn inhibits Golgi-to-ER retrograde trafficking. As a consequence, chaperones harboring a KDEL motif and recycling between the ER and Golgi complex become depleted from the ER and accumulate in the *cis*-Golgi thereby leading to Golgi complex fragmentation [6]. Another neurodegenerative disease that also has at its core changes in membrane trafficking is proximal spinal muscular atrophy. In normal cells, the *SMN1* gene encodes the survival of motor neuron 1 protein, involved in snRNP assembly and which has been shown to move along the neuronal axon in granules. However, in proximal spinal muscular atrophy, which results from the loss of expression of *SMN1*, a global blockade of granule export from the *trans*-Golgi network is observed, and granule accumulation occurs. This ultimately results in decreased levels of SMN1 in neurites and corresponding growth cone defects [7].

Other cell and tissue types are also sensitive to the blockage of trafficking from the Golgi complex. For example, dyschromatosis universalis hereditaria is a disorder characterized by the appearance of asymptomatic hyper- and hypo-pigmented macules in the skin. Recently three missense mutations have been identified in the ATP-binding cassette sub-family 8 member 6 (*ABCB6*) gene, which codes for a protein expressed in the epidermis and which is normally localized to endosome-like compartments and to the dendrite tips. All mutations led to retention of the protein in the Golgi complex [8], but it is unclear currently as to whether this is due to a general block in membrane trafficking or if the mutations affect the targeting of this protein only.

## 3. When Shape Changes

One possible consequence of mutations in Golgi complex proteins is that they cause gross changes in the morphology of the entire organelle and protein mislocalization, which together result in functional problems such as impairment of glycosylation. However, it is still unclear as to the importance of the physical integrity of this organelle with respect to its various functions. For example, live cell imaging experiments have shown that in the presence of the microtubule disrupting agent nocodazole, which also fragments the Golgi complex, secretory traffic between the ER and Golgi complex is initially retarded [9], although with prolonged nocodazole treatment transfer of material from ER to Golgi is restored [10]. By contrast, in nocodazole-treated cells, Golgi complex to cell surface transport still occurs with normal kinetics [11]. Nevertheless, there are examples where morphological changes to this organelle can have functional consequences and linkage to disease can be found. In a cellular model of Parkinson’s disease in PC12 cells, the Golgi fragmentation that is observed has been found to be dependent on the expression levels of certain members of the Rab family of small GTP binding proteins [12]. At the cellular level, Parkinson’s disease is associated with the accumulation of the pre-synaptic protein α-synuclein, and a block in ER-Golgi transport [13]. In this new study, fragmentation was correlated with changes in levels of RAB1, RAB2, RAB8 and the SNARE fusion protein syntaxin-5 (STX5), but that the phenotype could be rescued when RAB1 and RAB8 were overexpressed and RAB2 and STX5 were depleted [12]. All these proteins are well established early secretory pathway machinery molecules, however this study indicated that the Golgi fragmentation occurred prior to α-synuclein aggregation and might indeed promote it, thereby leading to the formation of the inclusion bodies. Other Rab proteins of the Golgi complex are also implicated in disease; mutations in *RAB33B* have recently been shown to lead to Golgi swelling and fragmentation in Smith-McCort dysplasia [14]. Although further experiments are still required, imaging approaches focusing on Rab behavior in living cells [15] are highly likely to further our understanding of these key membrane traffic molecules in disease.

**Figure 1:**
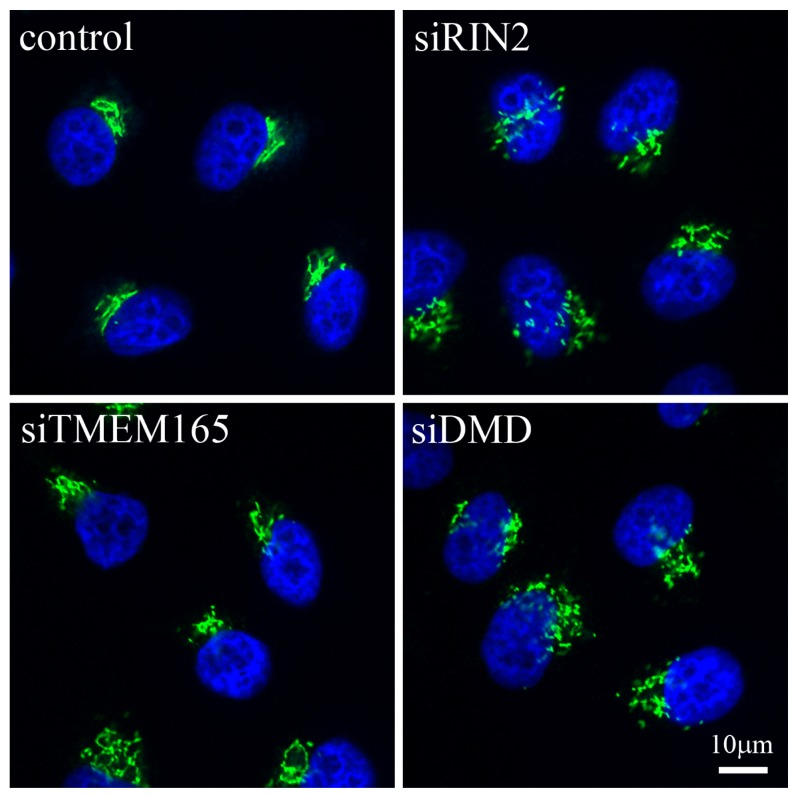
Immunofluorescence showing gross changes in Golgi complex morphology in response to the down-regulation of particular proteins. HeLa cells were incubated with RNAi reagents targeting *RIN2*, *TMEM165* or *DMD* and incubated for 48 h. Cells were fixed and stained for the Golgi matrix protein GM130 (green) and the nucleus (blue). A normal compact juxta-nuclear Golgi complex can be seen in control cells, whereas the cells depleted for the three proteins of interest show varying degrees of change in Golgi complex morphology. Scale bar corresponds to 10 μm.

Proteins that modulate the function of members of the Rab family of proteins have also been involved in the pathogenesis of Golgi-related diseases. The Ras and Rab interactor 2 (RIN2) protein, a guanine nucleotide exchange factor for the early endocytic pathway regulator RAB5 [16], has been found to be at the heart of two related connective tissue disorders, namely macrocephaly, alopecia, cutis laxa and scoliosis (MACS) syndrome and the RIN2 syndrome [17,18]. In these diseases, two independent frameshift mutations that lead to a decrease in mRNA expression levels and absence of the protein in patients’ fibroblasts were described. In the case of the MACS syndrome, the authors report an accumulation of vacuoles in the Golgi, but no other changes to the organelle [17]. By contrast, in RIN2 syndrome, dilation of the ER and rarified and dilated Golgi cisternae were observed [18]. In the latter, impairment of trafficking from the ER to the Golgi complex and from the Golgi to the plasma membrane were also detected, demonstrating that the consequences of the mutations affect the organelle in more than one way. Techniques such as RNA interference (RNAi) in cultured mammalian cells should further help us understand the cellular consequences of changes in proteins such as RIN2. At the macro level, immunofluorescence studies clearly show that the depletion of RIN2 indeed affects the overall morphology of the Golgi complex, when compared to control cells (Figure 1). Deeper analysis of data from large-scale RNAi studies looking at secretory pathway function [19] and Golgi morphology [20] will inevitably provide more candidates associated with Golgi morphology and function, which may be linked to disease.

One final recent example reporting a potential link between a Golgi complex protein and disease is centered on the previously uncharacterized transmembrane protein TMEM165. This protein may be involved in proton/calcium transport, and has been found to be linked to congenital disorders of type II glycosylation [21–23]. Four different disease-causing mutations that lead to decreased protein expression have been identified in *TMEM165*, one of which results in alternative splicing of the mRNA with no expression of the mutant protein [21]. The wild-type protein was found to localize to the Golgi complex and also to the endosomal-lysosomal system, whereas each of the four mutants analyzed were found in different locations in the cell [23]. The fact that the Golgi complex appears to be swollen and that the *trans*-Golgi network is fragmented has been suggested to indicate that this protein is involved in maintenance of the morphology of the organelle [21]. Cellular depletion of TMEM165 using RNAi appears only to have a mild effect on the Golgi complex at the macro level (Figure 1).

## 4. When Glycosylation Is Impaired

The literature regarding diseases of glycosylation has been extensively reviewed elsewhere [24–27]. Nevertheless, since almost all proteins passing through the secretory pathway undergo some form of glycosylation, it is highly likely that there are still more diseases associated with this process to be discovered. Defects in protein glycosylation lead to diseases of varied etiology ranging from muscular [28] to multi-system disorders such as the conserved oligomeric Golgi (COG) complex-dependent human glycosylation disorders [26]. Of note is the fact that the detection of these diseases is often serendipitous and those identified so far are primarily severe syndromes some of which are even fatal. Although many glycosylation disorders are caused by loss-of-function mutations in glycosyltransferases or sugar transporters, mutations have also been described in proteins associated with the COG complex, a multi-protein assembly involved in vesicle trafficking processes at the Golgi complex.

A small number of diseases have been described that result from a change in the acidification of the Golgi complex. Increases in the intra-luminal Golgi pH disrupts morphology of the organelle, affects protein and lipid processing and glycosylation, and alters cargo sorting [29]. In Angelman syndrome, a severe and rare neurodevelopmental disorder, it has been reported that the lack of ubiquitin protein ligase E3A (*UBE3A*) expression leads to an increase in the Golgi pH, which in turn causes osmotic swelling of the Golgi cisternae with a concurrent reduction in protein sialylation, a process highly dependent on pH [30]. Cutis Laxa type II and wrinkly skin syndrome, diseases that affect the connective tissue, and in particular the skin, are caused by mutations to *ATP6V0A2*, a vacuolar ATPase that is localized to the Golgi complex and is involved in its acidification [31,32]. Although the exact mechanisms by which these mutations cause the disease still remain to be established, one can speculate that perturbations to the intra-luminal Golgi pH will inevitably lead to abnormal protein glycosylation and possible aberrant Golgi trafficking [33].

Interestingly, there are also likely to be further links discovered between defects in glycosylation and aberrant Golgi complex morphology. This is because in order for glycosylation events to be tightly regulated, distinct classes of glycosylation enzyme need to be physically partitioned from one another, and therefore any morphological rearrangements of the individual cisternae may compromise this situation. As described above, mutations in *TMEM165* induce morphological changes in the Golgi complex, however in addition the authors of these studies also describe a reduction in protein glycosylation [21,22]. Since the function of numerous proteins involved in bone metabolism or belonging to the extracellular matrix can be affected by abnormal glycosylation, impairment of this process provides one explanation for the skeletal manifestations observed in the disease [34].

## 5. When Function Is Lost

Finally, we address the issue of the effects that can be caused when the expression of a Golgi-resident protein is lost due to mutation. Perhaps the most well-known case is in Duchenne muscular dystrophy, where the dystrophin (DMD) protein is not expressed leading to aberrant Golgi organization [35] (Figure 1). However, because this particular condition has been recently reviewed elsewhere [36], we will focus our attention on other Golgi complex proteins, the loss of which induces disease.

The first example is RAB33B, a member of the Rab family of small GTP binding proteins, which is localized to the Golgi complex and has been proposed to be involved in retrograde traffic back to the ER [37,38] and also more recently has been connected to autophagy [39,40]. Two missense mutations in the GTPase domain of RAB33B have been identified in patients with two different skeletal dysplasias. Interestingly in Dyggve-Melchior-Clausen disease no changes were detected at the level of the Golgi complex [41], whereas in Smith-McCort Dysplasia reduced RAB33B protein expression levels were correlated with a swollen and fragmented Golgi complex [14]. Another observation is that the mutation found in Dyggve-Melchior-Clausen disease occurs at a lysine residue that is conserved in *RAB33B* orthologs across all species [41]. In fact, this lysine residue is found in the GTP binding pocket of all Rab family members thereby demonstrating the importance of this residue to the function of the protein. Of note is the fact that both diseases can also be caused by mutations to another Golgi-resident protein, dymeclin [42], and so further studies will be needed to establish for these diseases the relationship between this protein and RAB33B.

A second example of loss of expression of a Golgi protein is also seen with another member of the Rab GTPase family. In X-linked Mental retardation associated with autism, epilepsy and macrocephaly one study has found two different loss-of-function mutations in *RAB39B*. This particular Rab protein was originally described as being expressed in a wide variety of tissues [43], although this more recent study highlights its role in the Golgi complex of neuronal cells [44]. Both reported mutations result in loss of protein expression in neurons, which in turn leads to an altered number and morphology of neurite growth cones and reduction of presynaptic buttons [44]. This work is important not only with respect to the elucidation of the disease mechanisms, but is also useful to begin to understand the function of RAB39B at the cellular level. Indeed the results suggest that this protein is somehow involved in the development of neurons and human intellectual ability.

Another example also with potential functional links to the Rab family is *SCYL1BP1/GORAB*, a RAB6-interacting golgin. Mutations in this gene were found to cause Gerodermia osteodysplastica [45,46]. Most of the mutations identified in this gene lead to a loss of protein expression, however in one affected family the mutation still led to protein expression but the function was lost [46]. In all cases no detectable changes in Golgi complex morphology were found.

One final example is *GOSR2*, a Golgi-resident Qb-SNARE involved in intra-Golgi trafficking, which has been linked to “North Sea” progressive myoclonus epilepsy, a progressive neurodegenerative disease associated with skeletal deformities [47,48]. The authors of this study describe a missense mutation in a glycine residue conserved across species and present in all three *GOSR2* isoforms. Although this mutation still leads to protein expression, it fails to localize to the Golgi complex. In addition, studies performed with a yeast strain lacking the *GOSR2* ortholog demonstrate that overexpression of the mutant protein is unable to rescue the phenotype and the cells die, suggesting that the disease-associated mutation is equivalent to a loss-of-function mutation [47].

## 6. Conclusions

The last five years have seen a significant number of human diseases being linked to Golgi complex proteins (Table 1). Not only are these discoveries important from a health perspective, but also they provide an additional avenue of insight for us to understand the function of this organelle at the molecular and cellular level through to the organismal level. Although the list of proteins that we have assembled here is far from complete, it is becoming clear that the number of disease-related proteins linked to Golgi homeostasis may eventually rival those known to be important for diseases associated with other key organelles such as the ER [49,50] and lysosomes [51].

Many of the proteins that we highlight in this review are unsurprisingly associated with trafficking through the Golgi complex (e.g., the Rab family of proteins), while others play a role in maintaining the homeostasis of the organelle (e.g., ATP6V0A2). One challenge however, is to unravel the cause and effect of each cellular phenotype. For example, mutation of a transporter of the Golgi complex may affect the luminal pH, which in turn affects the glycosylation efficiencies of particular enzymes, which then ultimately may alter the flux of all proteins passing through the organelle. Conversely, defects in a proteins associated with trafficking may cause morphological changes to the organelle, which in turn may affect specific biochemical or enzymatic reactions requiring a discrete micro-environment. To date, only in a few cases has it been possible to precisely link the protein to a particular phenotype, whereas in many more cases how the etiology of the disease is derived from the cellular events remains to be established. Nevertheless, the identification of disease causing mutations in specific proteins is very useful to physicians as it allows them to perform more accurate diagnoses and ultimately provide better advice to affected families. However, there is still much to be done, as in the majority of the reports that we highlight here, only a limited amount of molecular and cellular information has been gathered. Indeed for many of these proteins now linked to disease we still do not know their fundamental biochemical characteristics at the cellular level—for example are they degraded more rapidly than the wild-type protein, do they aggregate, or are protein-protein interactions impaired? Only a more complete knowledge of the molecular basis of the disease will ultimately allow appropriate therapies to be developed. Another obstacle to the elucidation of the molecular mechanisms of many of these diseases is that they affect organ systems that are challenging to work with, such as the central nervous system. In addition, the limited availability of patient tissues (as many of these disorders are rare) is also problematic.

In the forthcoming years it is to be expected that further disease causing mutations will be identified in proteins that physically and functionally are associated with the Golgi complex. More than 100 years after the discovery of this organelle, it is only now that we are beginning to realize the importance of the Golgi complex in cells throughout the human body, and the critical role that it plays at the heart of the endomembrane system.

## Figures and Tables

**Table 1:** Proteins of the Golgi complex associated with disease.

Affected gene	Disease	Primary clinical manifestation	Comments/Cellular effect/Reference
*ABCB6*	Dyschromatosis universalis hereditaria	Skin disorder	Mutation leads to retention of the protein in the Golgi [8]
*ATP6V0A2*	Cutis laxa	Connective tissue disorder	Mutations lead to abnormal glycosylation of serum proteins (CDG-II) and impairment of Golgi trafficking [31–33]
*ATP7A*	Menkes disease, occipital horn syndrome	Neurodegeneration and connective tissue disorder	Protein is localized to the TGN and is essential for copper metabolism. A wide variety of reported mutations affect its localization and trafficking pathways through the Golgi [52]
*ATP7B*	Wilson disease	Hepatic and neurological disorders	Protein is localized to the TGN and is essential for copper metabolism. A wide variety of reported mutations affect its localization and trafficking pathways through the Golgi [53]
*COG1*, *COG4*, *COG5*, *COG6*, *COG6*, *COG7*, *COG8*	Congenital disorders of glycosylation	Multi-system disorders	Typically reduced levels of the COG member occur, leading to defects in glycosylation [26]
*DMD*	Duchenne muscular dystrophy	Muscular disease	Absence of DMD leads to aberrant organization of the Golgi [35]
*FGD1*	Aarskog-Scott syndrome/faciogenital dysplasia	Skeletal and genital abnormalities	Localized to the TGN with mutants causing a reduction in trafficking from the Golgi [54]
*GOSR2*	“North Sea” progressive myoclonus epilepsy	Neurological disease	Mutant protein fails to localize to the *cis*-Golgi [47,48]
*PLP1*	Pelizaeus-Merzbacher disease	Neurological disease	Mutation in *PLP1* leads to depletion of ER Chaperones with a KDEL motif and Golgi fragmentation [6]
*RAB1*, *RAB2*, *RAB8*, *STX5*	Parkinson’s disease	Neurological disease	Altered expression of the proteins leads to Golgi fragmentation [12]
*RAB33B*	Dyggve-Melchior-Clausen disease	Skeletal dysplasia	Missense mutation leads to decreased protein expression [41]
*RAB33B*	Smith-McCort Dysplasia	Skeletal dysplasia	Missense mutation leads to lower protein expression and swollen and fragmented Golgi in many cells [14]
*RAB39B*	X-linked mental retardation associated with autism, epilepsy and macrocephaly	Neurological disease	Loss of function mutations in *RAB39B* lead to altered number and morphology of neurite growth cones and reduction of presynaptic buttons [44]
*RIN2*	Macrocephaly, alopecia, cutis laxa and scoliosis (MACS) syndrome	Connective tissue disorder	Loss of function mutation leads to presence of vacuoles in the Golgi [17]
*RIN2*	RIN2 syndrome	Connective tissue disorder	Loss of function mutation leads to dilation of ER, and rarified and dilated Golgi cisternae [18]
*SCYL1BP1/GORAB*	Gerodermia osteodysplastica	Connective tissue disorder	Loss of function mutation [45,46]
*SMN*	Proximal spinal muscular atrophy	Neurological disease	Decreased expression of *SMN* leads to accumulation of SMN granules in the *trans*-Golgi network and a global blockage of granule secretion [7]
*TMEM165*	Congenital disorder of glycosylation type II (CGD-II)	Psychomotor retardation and bone dysplasia	Mutation leads to lower protein expression and altered subcellular localization with overall Golgi swelling and fragmented *trans*-Golgi network. Decreased protein glycosylation is also observed [21–23]
*UBE3A*	Angelman syndrome	Neurodevelopmental Disorder	Loss of protein expression leads to an altered Golgi morphology and pH, which is associated with a reduction in protein sialylation [30]
